# Research on Migrant Works' Concern Recognition and Emotion Analysis Based on Web Text Data

**DOI:** 10.3389/fpsyg.2021.741928

**Published:** 2021-08-31

**Authors:** Zhijie Dou, Zixuan Cheng, Dongmei Huang

**Affiliations:** ^1^Shool of Management, Changchun University, Changchun, China; ^2^School of Electronic and Information Engineering, Changchun University, Changchun, China; ^3^Department of Psychology, School of Philosophy and Sociology, Jilin University, Changchun, China

**Keywords:** migrant workers, concerns, emotion analysis, Word2Vec, Bi-LSTM, CNN

## Abstract

Based on the characteristics of convenience, autonomy, and equality, online self-media has become an important way for contemporary migrant workers to observe the world, understand society, examine themselves and express their demands. On the basis of the analysis of the domestic migrant works' concerns and their emotion analysis, we crawl data on Weibo about migrant works' topics as the basic corpus of migrant works' concerns, and then uses a combination of TF-IDF and Word2Vec methods to construct a recognition model of migrant workers' concerns. We found that wages, children's education, medical care and returning home are the main concerns of migrant workers. Meanwhile, further emotion analysis of the migrant works' concerns of using a deep learning model fused with Bi-LSTM and CNN was conducted. The results show that the proportion of negative emotion such as worries, complaints and impetuosity was significantly higher than that of other positive and neutral emotion like encourage and comfort. And the time when the negative emotion are concentrated is significantly related to the social events that occur in the corresponding time period. On the one hand, it shows that the concerns and emotion of migrant workers can be effectively observed and predicted through web text data. On the other hand, it also shows that the core well-being issues of migrant workers in the process of urban integration have not been effectively solved, and the government and relevant departments need to take targeted measures and give priority attention.

## Introduction

Two billion farmers stand at the entrance of industrial civilization. This is the main problem posed to the social sciences in the second half of the 20th century. Since the mid to late 1980s, driven by marketization, industrialization and urbanization, a large number of rural labor forces in our country have transferred to cities, and a new social class called the migrant worker class has gradually formed. As the product of the era of the dual oppositional structure of urban and rural household registration, it has attracted widespread attention from the government, media, academia and even the society. It has become one of the important topics in the localization of social sciences and has achieved fruitful research results. However, most of the existing research focus on the four aspects of group characteristics summary (Looyestyn et al., 2018), urban integration exploration (Mikulincer et al., 2005; Chou, 2009), mental health evaluation (Leavey et al., 2007; Li et al., 2007) and social identity (Zerubavel, 2000; Sb et al., 2020). The problems of migrant works' emotion have not yet obtained enough attention. As the key link between micro social reality and macro social reality, almost any aspect of human cognition, behavior and social organization is driven by emotion. Migrant workers are the emotional subjects with flesh and blood, joy and sorrow, and they also have spiritual demands and emotional needs. Therefore, to truly understand the living conditions and behavioral logic of migrant workers, especially migrant workers in the new era, emotion are just an indispensable link. In the study of migrant workers, if scholars ignore or simply pay attention to migrant works' emotion instead of digging into them, they cannot fully explain why migrant workers make some objectively apparently irrational decision-making actions, such as publicly beating leaders. At the same time, they cannot understand why migrant workers form certain mentalities and concepts, such as thinking that college students of the same age are naive. As a result, it is not conducive to grasping the interactive relationship between the micro-fortunes of migrant workers and the macro-structure of society. In addition, the process of migrant workers' integration into the city is the process by which farmers move from a rural society that characterizes traditional civilization to an urban society that characterizes modern civilization. It is also a resocialization mechanism. Existing studies have shown that social integration can be seen from two dimensions, one is behavior fusion and the other is emotion fusion (Kisar Koramaz, 2014). The close relationship among emotion, social structure and cultural environment makes the research of migrant workers' emotion complementary to urban integration and social identity. Therefore, the emotion adaptation efforts of migrant workers in the process of integrating into the city deserve an academic response. Especially the new generation of migrant workers, as a product of the internal structure iteration of the rural migrant labor group for more than 30 years, their emotion experience is more worthy of our attention. Compared to their parents who are based on survival rationality during their working careers and have limited levels and scope of emotional generation.

With the popularization and development of information technology, social media platforms such as Weibo, Moments, Zhihu, and Post Bar have become new ways for migrant workers to obtain information, express their opinions, express their emotion, and clarify their standpoints. According to public statistics, as of December 2020, the number of Chinese Internet users has reached 989 million. In the professional structure of netizens, laborers in agriculture, forestry, animal husbandry, fishery and rural migrant workers accounted for 15.3 and 11.4%, respectively, ranking the third and fourth. Agricultural laborers and migrant workers are important constituents of Chinese netizens. They will publish text comments with personal emotion on related events or topics, and these subjective texts often contain great value. By mining and analyzing the web text data, it is possible to identify the migrant works' concerns and emotion. This will help government departments to further understand issues of common concern for migrant worker and their mental status. In this way, targeted policies can be designed to better meet their demands. In view of this, the paper combines TF-IDF and Word2Vec to construct a recognition model of migrant workers' concerns, and mines the main concerns of migrant workers contained in the web text data. Then we use a deep learning model that combines bidirectional long and short-term memory network (Bi-LSTM) and convolutional neural network (CNN) to perform emotion analysis on the concerns of migrant workers.

Finally, The structure of the paper is as follows:

Part 1: The first part introduces the research background and value of the paper.

Part 2: Introduce the related research work of the thesis, and summarize the innovative points of the paper.

Part 3: Establish a model of migrant works' concerns recognition and emotion analysis based on web text data.

Part 4: Empirical results and analysis are conducted to prove the effectiveness of the model used.

## Backgrounds

### Concerns Recognition

The migrant works' concerns can reflect the issues that migrant workers generally concern and their ideological status, which are an important basis for decision-making by the government and other relevant departments. In the past, most of the research on migrant workers was based on questionnaires, on-site interviews or discussions to collect data. It not only wasted time and energy, but the number of samples obtained was also very limited, which could not fully cover the concerns of migrant workers. According to Goffman's theater theory, our daily life and social activities can be compared to a dramatic performance. The performance area is divided into the front stage and the back stage. The front stage can be seen as a personal image based on the script, rather than a real personal image. In other words, in face-to-face communication, migrant workers may hide their true feelings, which makes it impossible for us to directly understand their concerns.

In recent years, with the help of new media represented by the mobile Internet, massive amounts of network data have shown an eruptive growth, and a large number of researches on the recognition of concerns on online social networks have emerged. Patil and Gupta (2016) and Chen et al. (2014) established a work process that combines qualitative analysis and large-scale data mining technology by comprehensively analyzing the literature on mining user social media data. Gou and Gaikwad (2016) dig out students' data from Twitter, and use qualitative analysis to find problems in students' daily learning life, such as heavy learning burden, lack of social skills, etc. In recent years, Patil and Kulkarni (2018) have further used Twitter API to obtain data on students' concerns, and classified the data into six major categories. And then the Memetic algorithm is used to classify the data. Compared with Bayes and ID3, the experimental results prove that the Memetic algorithm is more effective. At present, the topic recognition and tracking of online social network has become one of the research hotspots in the field of data mining, which has attracted the attention of more and more scholars, and has achieved fruitful research results (Vo and Ock, 2015; Bicalho et al., 2017). This also provides a lot of reference for us to dig out the concerns of migrant workers.

### Emotion Analysis

Text emotion analysis, also known as emotion analysis or opinion mining, is a research hotspot in the field of text analysis. It mainly uses the feature mining or learning of the contextual content information of the text to judge its emotional tendency, which is a text classification task that correlates emotion. The text to be analyzed can be content such as network comments, articles, and microblogs. In the current Internet era, news media platforms and various social media platforms generate a large number of user comments every day. Automatic emotion analysis of the data is helpful to understand the user's attitude toward specific events and topics, and to grasp public opinions so order to make corresponding decisions. Generally speaking, the methods of text emotion analysis can be divided into methods based on emotion dictionary, methods based on statistics, and methods based on deep learning.

The method based on the emotion dictionary needs to build an emotion dictionary composed of emotion words, and then use semantic rules to calculate the semantic similarity to judge the emotion tendency of the text. For example, Chesley et al. (2006) uses verbs and adjectives to construct emotion templates to obtain the emotion information of the text and achieve emotion classification. Joshi et al. (2011) uses the artificially annotated Twitter emotion information and traverses the emotion score in the text to judge the emotion polarity. Turney (2002) uses Pointwise Mutual Information (PMI) between words to classify text emotion. In summary, the methods based on emotion dictionary often relies heavily on the construction of emotion dictionaries and the design of rules, which has high labor costs and cannot identify text emotion information outside of the rules.

With the rapid development of machine learning, many scholars apply machine learning algorithms to text emotion analysis. Generally, emotion analysis methods based on machine learning mainly include support vector machines, decision trees, and naive Bayes. In emotion analysis tasks, the machine learning algorithms achieve a good classification effect by extracting information from the data set and constructing features. For example, in 2009, Boiy and Moens (2009) trained a variety of machine learning models and cascaded them into the final emotion classification model to solve the problem of multilingual emotion analysis. This method effectively compensates for the shortcomings of a single algorithm through the combination of different models. In 2013, Mikolov et al. (2013a) used a support vector machine (SVM) classification model that combines diversified features to complete the emotion classification task of Weibo text. The model expresses the characteristics of different types of words, which can make full use of the emotion information in the sentence and improve the effect of emotion classification. The above methods of text emotion analysis based on emotion dictionary and machine learning not only requires manual construction of high-quality emotion dictionary and feature engineering, but also consumes a lot of human resources, so it is difficult to be widely used in text emotion analysis tasks in open fields.

In recent years, due to the strong feature extraction capabilities of DNN, a variety of methods based on DNN have been widely used in emotion analysis and text classification. Unlike the traditional machine learning methods whose features are often sparse, deep neural network methods convert text into dense vectors and obtain high-level representations of text for classification. For example Vieira and Moura (2017) proposed a convolutional neural network based on sentence dimensions. This method sets different hyperparameters for different emotion types for training, so as to realize the task of text emotion analysis in sentence dimension. Since then, many learners have conducted more in-depth research on text emotion analysis methods. Yoon and Kim (2017) used the CNN-BiLSTM model of multi-channel word embedding for text emotion analysis. It can capture the high-level semantic relationships and long-term dependencies in the text, and has achieved good results on the emotion analysis data set provided by Twitter. Chen et al. (2018) proposed a Multi-Channel Information Crossing (MIX) model for text emotion analysis. MIX compares text fragments of different granularities to form a series of multi-channel similarity matrices, and cross-use with another set of carefully designed attention matrices to extract richer sentence features. The above text emotion analysis methods based on deep learning improve the accuracy of text emotion analysis by extracting semantic features of various sentence dimensions.

Based on the above research, we further study the concerns and emotion analysis of migrant workers based on the web text data. The related research work is as follows:

(1) First, construct a data set of the migrant works' concerns. At present, there is no public data set on the concerns of migrant workers or corresponding methods for mining the concerns of migrant workers in China. We use web crawler technology to capture the data related on Weibo, and then store these pre-processed data in SQLSever2008 after filtering out invalid comments such as advertisements and spam links. After that, technologies such as web page cleaning, word segmentation, and stop word removal are used to pre-process the acquired data, thereby saving a lot of calculation content and improving the accuracy of the topic.

(2) Second, establish a classification model of migrant workers' concerns. Through qualitative analysis of the pre-processed data, a multi-level classification framework is concluded. The first level includes three major classes. The second level is the refinement of each major class, with a total of nine sub-classes. And the third level is the emotion classification for each sub-class, namely positive, neutral and negative emotion. Then use TF-IDF and Word2Vec methods to construct a classification model of migrant workers' concerns, and identify the concerns of migrant workers.

(3) Finally, the emotion analysis is carried out on the concerns of migrant workers. Based on the classification of migrant works' concerns, the paper uses a deep learning model that combines Bi-LSTM and CNN to perform emotion analysis on migrant works' concerns. So as to understand the migrant workers' thoughts and expectations in time, which can help the relevant government departments to solve the problems in a more targeted manner.

## The Model for Migrant Works' Concerns Recognition and Emotion Analysis Based on Web Text Data

**Figure 1:**
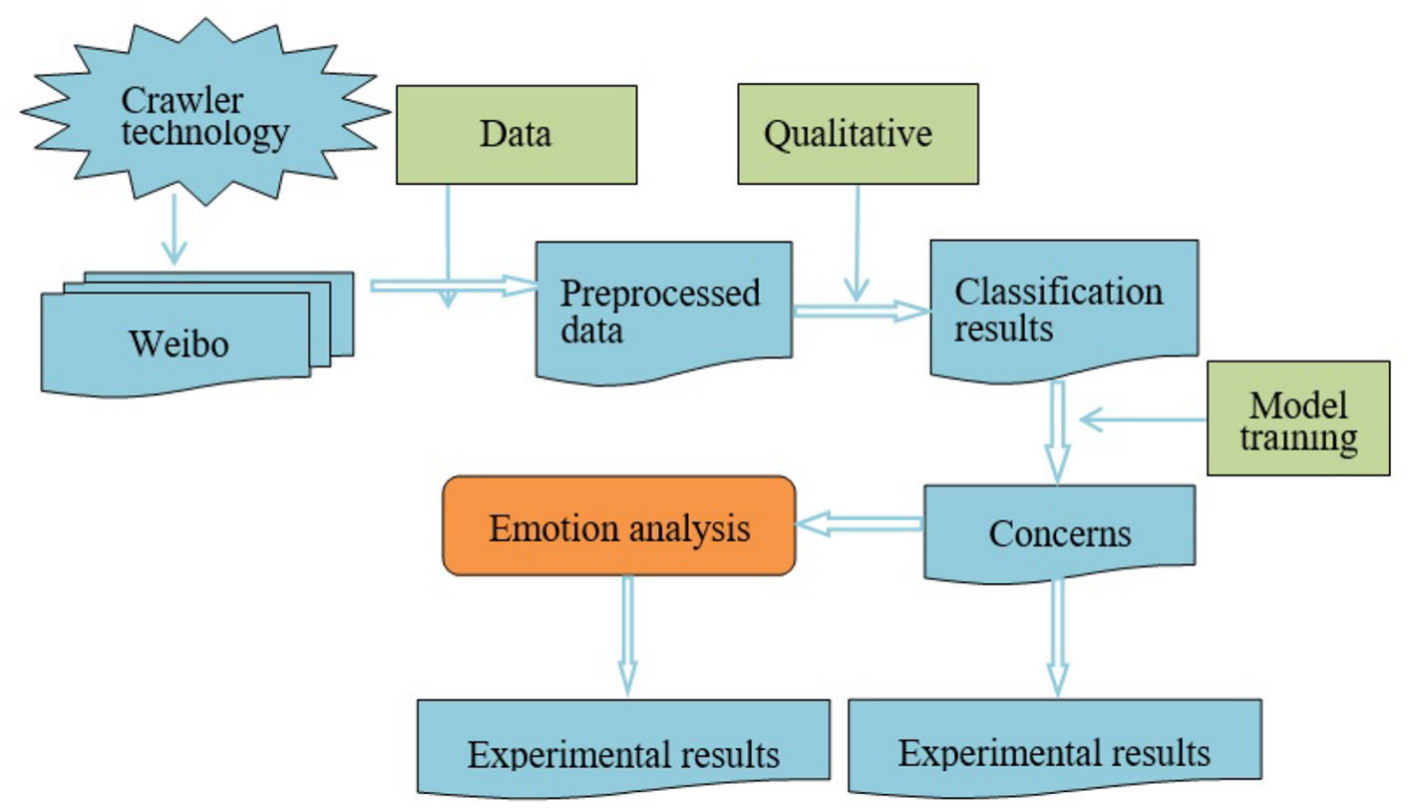
The frame chart of migrant works' concerns recognition and emotion analysis.

The research framework of the paper is shown in Figure 1, which is mainly divided into three parts. The first part is the collection and analysis of data sources. Firstly, we use crawler technology to obtain relevant data from social media and preprocess these data in turn to form a data source for migrant workers' concerns. The second part is to build a framework for migrant works' concerns. Based on the qualitative analysis of the acquired data, we found that migrant workers have different concerns at different stages, thus constructing a framework for migrant works' concerns. The third part is the construction of emotion analysis model. Different people will show different emotion tendencies toward the concerns. Based on the diversification of emotion, our paper establishes a method to analyze emotion tendencies. According to the results of emotion classification, we can clearly understand how migrant workers feel about different concerns.

### The Model of Migrant Works' Concerns

**Figure 2:**
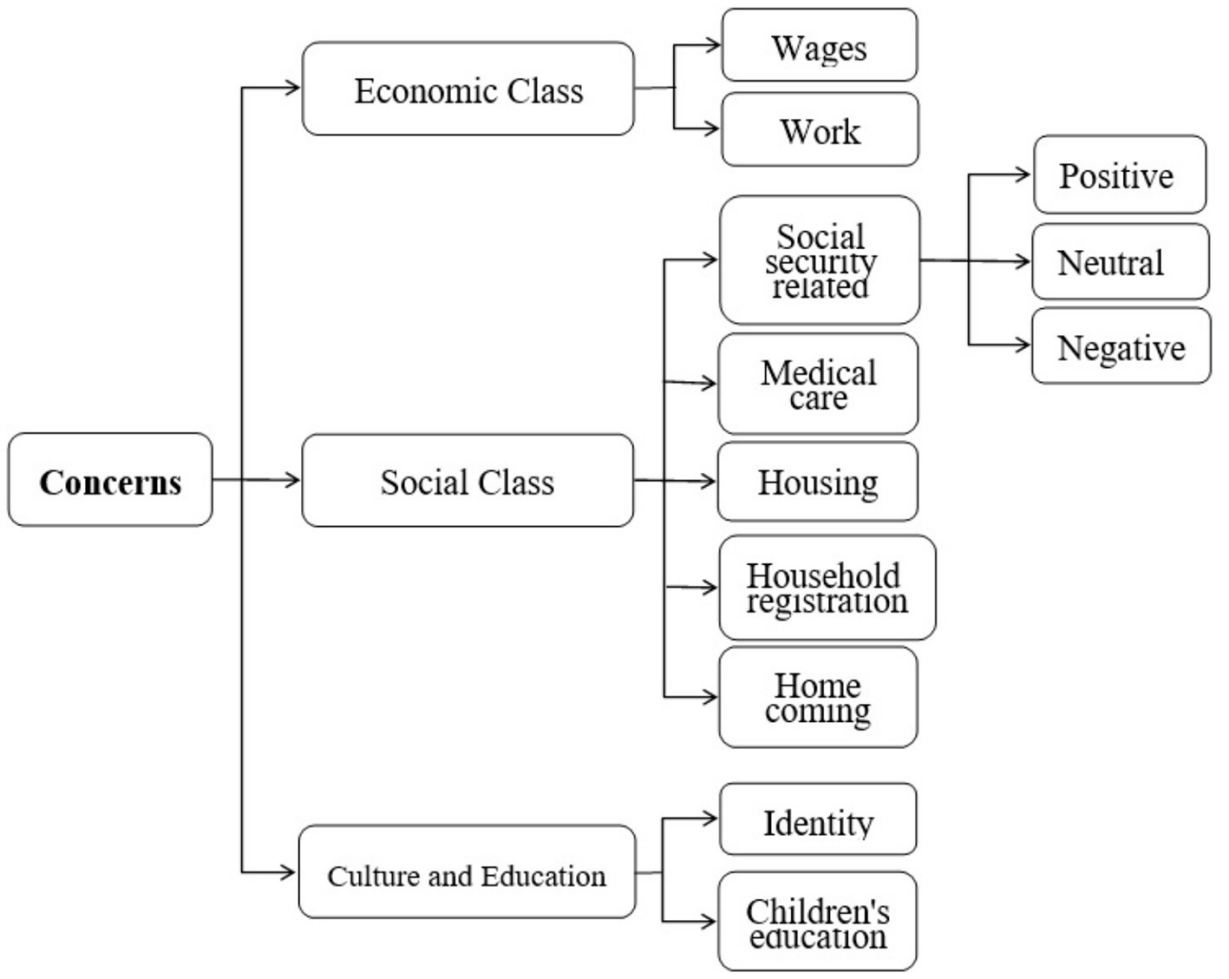
Classification framework of migrant works' concerns.

In the paper, since the pre-processed data has no pre-defined classes, we conduct an inductive analysis of all the data. And then a three-layer concerns classification framework is established. The first layer is mainly divided into three classes: economic aspects, social aspects and cultural and educational aspects. Secondly, the three major classes are further subdivided into nine sub-classes. The economy class is divided into two sub-classes: wages and work. The social aspect is further divided into five sub-classes, namely, social security related, medical care, housing, household registration and homecoming. Finally, the cultural education category is divided into two sub-classes, namely identity and children's education. In summary, the concerns of migrant workers are divided into three major classes and nine sub-classes. And the third level is the emotion classification for each sub-class, namely positive, neutral and negative emotion. The three-layer concerns classification framework is shown in Figure 2. We categorize the data collected from Weibo into the classes listed in Figure 2.

Combined with the classification framework of the migrant works' concerns, we further use a method based on the combination of TF-IDF and Word2Vec to classify the data sources collected from Weibo, and identify the concerns of migrant workers. The method fully integrates the semantic information of the sentence with the word vector and similarity calculation, and effectively improves the accuracy of classification.

We use the TF-IDF method proposed by Salton in 1988 to calculate the vocabulary weights, and the calculation is shown in formula (1):

(1) Wik=tf(k,i)×log(Nnk +0.01)∑k=1t(tf(k,i)×log(Nnk +0.01))2

Where, *W*_*ik*_ represents the TF-IDF value of the *k*th word in the *i*th post, and *tf(k,i)* is the frequency of the *k*th word in the *i*th post. *N* represents the total number of posts, *n*_*ik*_ is the number of posts containing the *k*th word, log(Nnk+0.01) is the IDF value of the *k*th word.

Then the Skip-gram model (Mikolov et al., 2013b) of the Word2Vec method is used to predict the context with a given input word, and its calculation formula is shown in equation (2).

(2) $$\begin{array}{l} P\left(w_{o}|w_{i}\right)=\frac{e^{U_{o}\cdot V_{t}}}{\sum_{j}e^{U_{j}\cdot V_{t}}} \end{array}$$

Where *V*_*t*_ represents the input vector, and *U*_*j*_ represents the output vector.

Finally, suppose that after the Word2Vec, each post *p*_*i*_ can be represented by μ keywords, namely *p*_*i*_ = {*wn*_1_, *wn*_2_, …, *wn*_μ_}(1 ≤ μ ≤ *K* × *M*). Define the class set as *F* = {*f*_1_, *f*_2_, …, *f*_9_}, where *f*_*j*_(*j* = 1, 2, …, 9) is the *j*th refined concern class. Define the posts of class *f*_*j*_ as {_*P*_*f*_*j*_−1_, *P*_*f*_*j*_−2_, …, *P*_*f*_*j*_ − |*f*_*j*_|_}_, |*f*_*j*_| is the number of posts contained in class *f*_*j*_. Then the formula for calculating the probability that the post *p*_*i*_ belongs to the class *f*_*j*_ is as follows:

(3) $$\begin{array}{l} P\left(p_{i},f_{j}\right)=\frac{\sum_{i=1}^{\mu }\sum_{i=1}^{\left|f_{j}\right|}tf\left(wn_{i},p_{f_{j}-i}\right)}{\sum_{k=1}^{9}\sum_{i=1}^{\mu }\sum_{i=1}^{\left|f_{k}\right|}tf\left(wn_{i},p_{f_{k}-i}\right)} \end{array}$$

### The Framework of Migrant Works' Emotion Analysis

**Figure 3:**
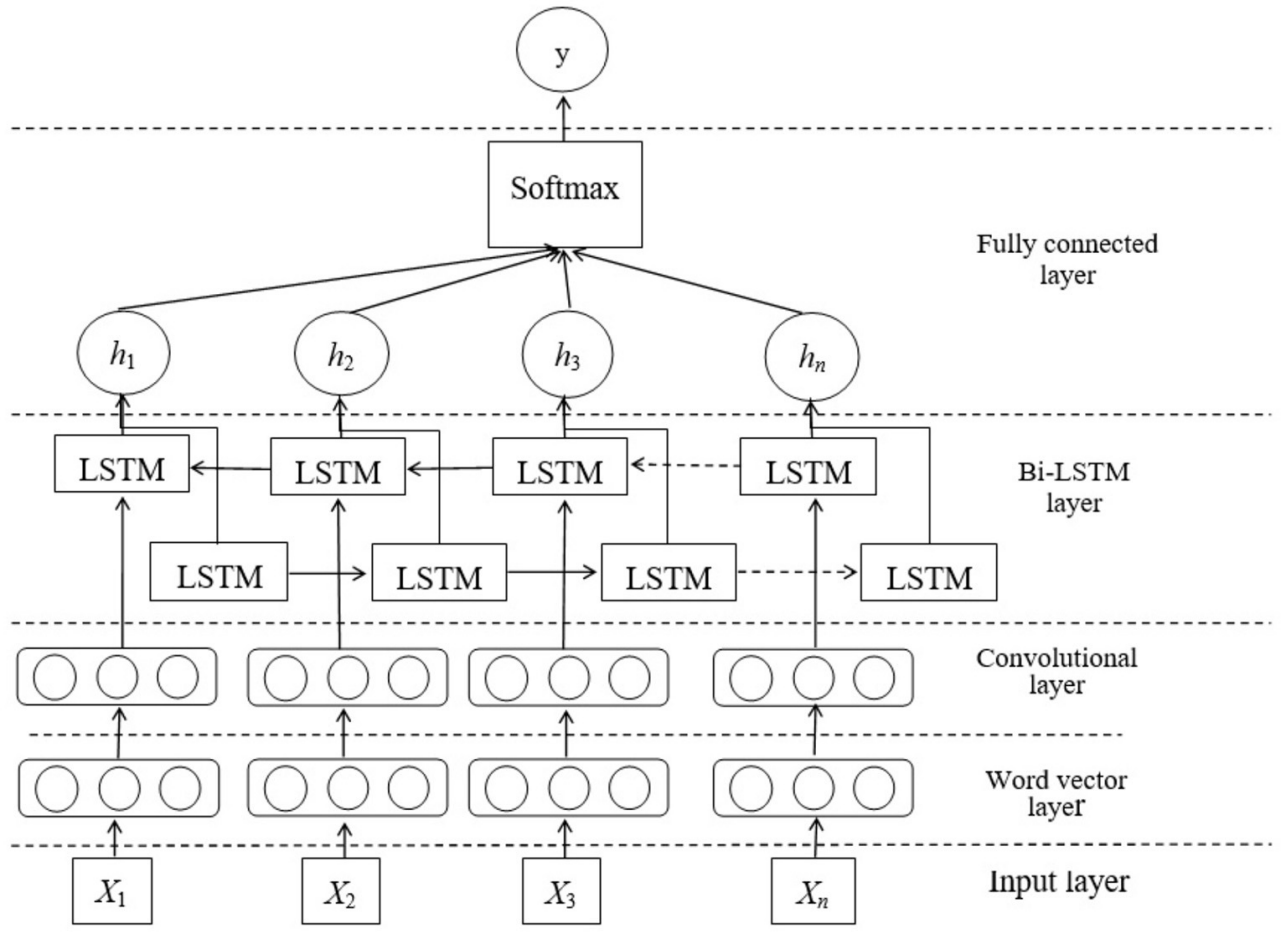
The network model of emotion analysis based on Bi-LSTM and CNN.

On the basis of recognizing the migrant works' concerns, we use a deep learning model combined with Bi-LSTM and CNN to analyze the emotion tendency of the migrant works' concerns. The network structure is shown in Figure 3.

As shown in the Figure 3, firstly, all posts of concerns will be represented by word vectors and input into the network after preprocessing. Secondly, the data will enter the convolutional layer and the memory layer, so as to make full use of CNN and Bi-LSTM, which is helpful for maintaining the text information and extracting features. Finally, the sofrmax classifier is used to obtain the classification results.

#### Word Vector Layer

First of all, the text of the input layer is segmented, and the word vector representation corresponding to each word in the text is obtained through Word2Vec (Lilleberg et al., 2015). Through the following formula, a word *x*_*i*_ can be converted into a word vector *h*_*i*_:

(4) $$\begin{array}{l} h_{i}=E_{w}v^{i} \end{array}$$

Where *E*_*w*_ represents the word vector matrix, and *v*^*i*^ indicates the size of the word vector. We use the method of randomly initializing the word vector, and it is constantly updated during the training process. Then the initial sentence will enter the convolutional layer in the form of word vector, namely *S*_*q*_ = {*h*_1_, *h*_2_, ⋯ , *h*_*n*_}.

#### Convolutional Layer

After the word vector layer, a sentence containing *n* words can be expressed as follows:

(5) $$\begin{array}{l} h_{0:n-1}=h_{0}⊕h_{1}⊕\cdots ⊕h_{n-1} \end{array}$$

The symbol ⊕ is a connector between two adjacent words. Generally, the vector *h*_*i*:*i*+*j*_ represents a series of word vectors *h*_*i*_, *h*_*i*+1_, ⋯ , *h*_*i*+*j*_. Each convolution operation contains a filter *w* ∈ *R*^*md*^ (where *d* represents the dimension of the vector). It can generate a new feature through a window containing *m* words. For example, a feature *c*_*i*_ can be generated by window *h*_*i*:*i*+*m*−1_:

(6) $$\begin{array}{l} c_{i}=f\left(w\cdot h_{i:i+m-1}+b\right) \end{array}$$

Here *b* ∈ *R* is a bias term, and *f* is a non-linear function similar to hyperbolic tangent. The input text can finally be expressed as:

(7) $$\begin{array}{l} c^{*}=\left[c_{0},c_{1},\cdots \;,c_{n-m}\right] \end{array}$$

#### Bi-LSTM Layer

LSTM network was originally used to solve the problem of vanishing gradient, and then many variant network structures based on LSTM were proposed. In the paper, we adopt a variant structure proposed by Graves et al. (2009), which can increase the weight of the peephole connection on the same memory module.

In particular, a recurrent neural network based on long and short-term memory has 4 main components: An input gate *i*_*t*_ with the weight matrix of *W*_*xi*_, *W*_*hi*_, *W*_*ci*_, *b*_*i*_. A forgetting gate *f*_*t*_ with the weight matrix of *W*_*xf*_, *W*_*hf*_, *W*_*cf*_, *b*_*f*_. An output gate *o*_*t*_with the weight matrix of *W*_*xo*_, *W*_*ho*_, *W*_*co*_, *b*_*o*_. All these dates will have a certain impact. Assuming that the current input *x*_*i*_, *h*_*i*−1_ has been generated in the previous step. Then, the current state of unit *c*_*i*−1_ determines whether to use the input *x*_*i*_, forgetting the previously stored memory. Finally output the generated state. These inferences can be proved by the following formulas:

(8) $$\begin{array}{l} i_{t}=\sigma \left(W_{xi}x_{t}+W_{hi}h_{t-1}+W_{ci}c_{t-1}+b_{i}\right) \end{array}$$

(9) $$\begin{array}{l} f_{t}=\sigma \left(W_{xf}x_{t}+W_{hf}h_{t-1}+W_{cf}c_{t-1}+b_{f}\right) \end{array}$$

(10) $$\begin{array}{l} g_{t}=\tanh\left(W_{xc}x_{t}+W_{hf}h_{t-1}+W_{cc}c_{t-1}+b_{c}\right) \end{array}$$

(11) $$\begin{array}{l} c_{t}=i_{t}g_{t}+f_{t}c_{t-1} \end{array}$$

(12) $$\begin{array}{l} o_{t}=\sigma \left(W_{xo}x_{t}+W_{ho}h_{t-1}+W_{co}c_{t-1}+b_{o}\right) \end{array}$$

(13) $$\begin{array}{l} h_{t}=o_{t}\tanh\left(c_{t}\right) \end{array}$$

Therefore, the generation of the current unit state *c*_*t*_ is determined by calculating the weight of the previous unit state and the current information generated by this unit. For many sentence-level processing tasks, it is necessary to consider contextual information. However, the standard LSTM network only considers the timing information when modeling the sentence and ignores the contextual information. The Bi-LSTM network expands the unidirectional LSTM network by introducing a second-layer network structure, and the hidden connections flow in the reverse time sequence. Therefore, Bi-LSTM can use the information of the context and ensure that the past and future information can be considered in the time series.

In the paper, we use the Bi-LSTM method to model the text sentences. As shown in Figure 3, the network contains two sub-networks to model text sentences before and after. The output of the *i*th word is shown in the following formula:

(14) $$\begin{array}{l} h_{i}=\left[\overset{\to }{h_{i}}⊕\overset{\leftarrow }{h_{i}}\right] \end{array}$$

#### Classifier

In this layer of the network, we use a softmax classifier to predict the label *y* of the text sentence *S* from a set of discrete categories *Y*. The classifier uses the hidden state c^*^ as input:

(15) $$\begin{array}{l} \hat{p}\left(y|S\right)=soft\;\max\left(W^{\left(s\right)}c^{*}+b^{\left(s\right)}\right) \end{array}$$

(16) $$\begin{array}{l} ŷ=\arg\max\hat{p}\left(y|S\right) \end{array}$$

The loss function is as follows:

(17) $$\begin{array}{l} J\left(\theta \right)=-\frac{1}{m}\overset{m}{\underset{i=1}{\sum }}t_{i}\log\left(y_{i}\right)+\lambda ||\theta |{|}_{F}^{2} \end{array}$$

Where *t* ∈ *R*^*m*^ is the representation of one-hot, *y* ∈ *R*^*m*^ represents the estimated probability of each class (*m* is the number of target classes), λ is the representation of L2 regularization parameter.

## Experimental Research and Analysis

The empirical research of the paper mainly includes two parts: Firstly, we analyze the crawled data and construct a multi-level classification framework. Then we use TF-IDF and Word2Vec methods to identify the concerns of migrant workers. Secondly, we use the deep network structure method fused with Bi-LSTM and CNN to judge the emotion tendency of migrant workers' concerns.

### Data Collection

As a social media and network platform for information sharing and exchange, Weibo is widely welcomed by migrant workers and has become the main platform for migrant workers in our country to share their experiences. They usually publish some articles or opinions in Weibo, which may be closely related to their work, life, family and social hot spots. Because of its social, freedom, anonymity and other characteristics, it is highly recognized by migrant workers, and it is also one of the important social platforms for migrant workers to show their emotion. In view of this, our paper chooses Weibo as the main source of data collection. Posts about migrant workers on Weibo cover a variety of topics, such as migrant workers' wages, recruitment, household registration and children's education. We use the Python technology to automatically retrieve the massive amount of scattered data on Weibo, and crawl the data as a corpus for concerns recognition and emotion analysis, with a total of about 20,000 lines. There are about 5,000 topic posts.

### Data Pre-processing

Since the collected data contains the content that is not relevant to our research, such as advertising spam, etc. Meanwhile, a large amount of irrelevant information will affect the accuracy of the concerns recognition and easily cause the analysis results to deviate from the topic. Therefore, data pre-processing is necessary. We use web cleaning technology, word segmentation, stop word removal and other related technologies to pre-process the crawled data to obtain effective data related to the research. It will save a lot of computing space and improve the accuracy of concerns classification.

### Experimental Results and Analysis

In the experiments, we use Precision which is commonly used in the text classification field, as the evaluation index. To show the effectiveness of the TF-IDF and Word2Vec methods used in the concerns classification, we also conducted comparative experiments with other classification methods. Table 1 shows the classification results of migrant workers' concerns based on different methods.

**Table 1:** The classification results of migrant workers' concerns based on different methods (%).

**Methods**	**Economic class**	**Social class**	**Cultural and educational class**
LDA	0.55	0.53	0.51
Word2Vec	0.62	0.60	0.65
TF-IDF+Word2Vec	0.74	0.73	0.75

Table 1 shows the classification results of migrant works' concerns in the three major classes of economy, society and culture. It can be seen from the results that compared with other methods, the classification accuracy of the TF-IDF+Word2Vec method is higher. It can also reflect the effectiveness of the method used in the paper.

**Figure 4:**
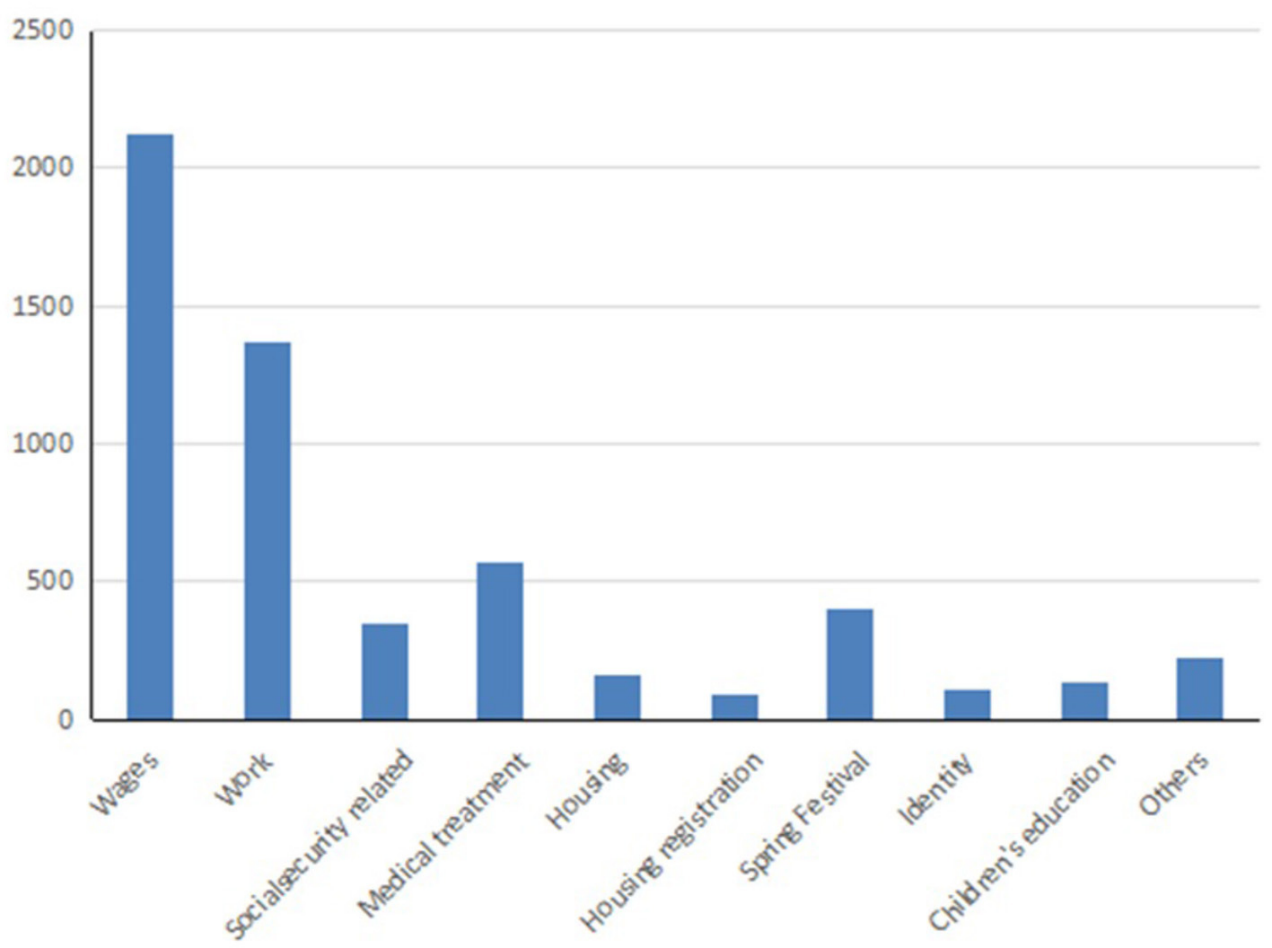
The histogram of distribution of migrant works' concerns.

In addition, we further visualized the data of migrant works' concerns. The histogram of the distribution of migrant works' concerns is shown in Figure 4. It should be noted that in the figure, the “other” class contains all posts that cannot be classified into any of the other nine classes. It can be seen from the figure that the migrant works' concerns mainly focus on wages, work, medical care, returning home during the Spring Festival and children's education, etc. It shows that the core well-being of migrant workers in the process of urban integration is still an important concern, and it is also a problem that relevant government departments and labor companies need to focus on and take effective measures to solve.

**Figure 5:**
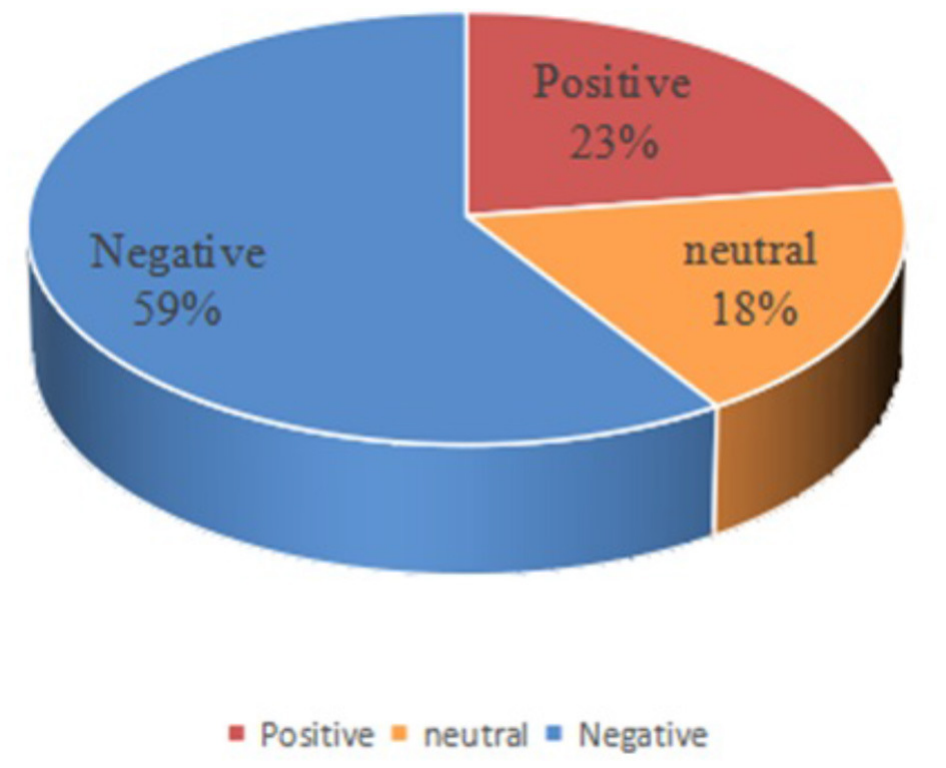
Distribution of emotion analysis of migrant works' “wages”.

According to the results shown in Figure 4, the topic of migrant works' wages is one of their most concerned concerns. Therefore, taking the data of migrant works' wage as an example, we use the method fused Bi-LSTM and CNN to perform emotion analysis on them. The text whose judgment result is “-1” is negative emotion, the text whose judgment result is “1” is positive emotion, and the text whose judgment result is “0” is neutral emotion. Then we got 482 positive texts, 1,257 negative texts and 386 neutral texts. The results are visualized as shown in Figure 5.

In the Figure 5, the yellow part represents neutral emotion, the red part represents positive emotion, and the blue part represents negative emotion. It can be seen from the figure that the proportion of negative emotion is far greater than that of neutral and positive emotion. Combining the results of visualization and emotion analysis, we found that on the issue of “wages,” migrant workers are generally concerned about topics such as “wage arrears, difficulty in asking for wages, and low wage income.” They express their worries, complaints, impetuousness and other negative emotion on social platforms, expecting to receive the attention of society and related departments, and receive comfort from others. The second largest proportion is positive emotion, which includes encouragement and comfort among migrant workers.

Through the above analysis, we can draw the following conclusions: On the one hand, due to anonymity, social platforms (such as Weibo) are ideal spaces for people with the same experience to communicate, as well as places to seek help. Compared with positive emotion, migrant workers prefer to post negative emotion on social platforms. On the other hand, the government departments should pay attention to the data in time and provide necessary assistance to help the migrant workers adjust their psychological state.

**Figure 6:**
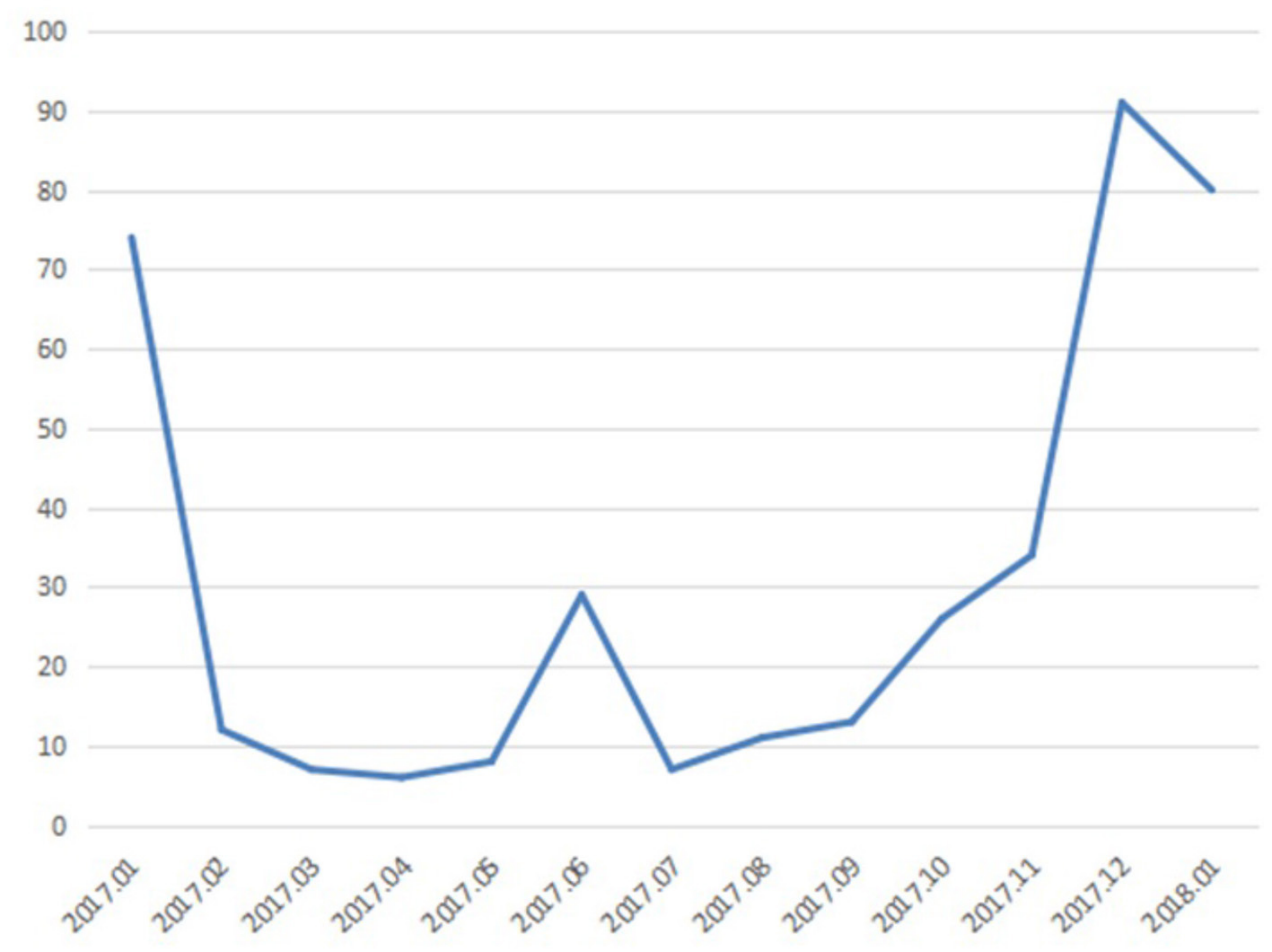
The time series distribution diagram of the negative emotion texts.

In addition, as an example, we also analyzed the time series distribution of negative emotion texts of migrant workers from 2017 to 2018. Set the time as the horizontal axis and the number of negative emotion texts as the vertical axis. Then a time series distribution diagram of the negative emotion texts is shown in Figure 6. In the early 2017, December 2017 to January 2018, the number of negative emotion texts was relatively higher. Especially in December 2017, the number of negative emotion texts increased sharply. Therefore, we speculate that there is some correlation between the negative emotion of the research group and the social events that occurred in the corresponding time period.

As shown in the Figure 6, we analyze and discuss the negative text content according to the time period. In the beginning of 2017 and the end of 2017, the topics like “migrant workers ask for wages” and “wage arrears” appear more frequently in the negative emotion text. This shows that the research group pays more attention to “wages” in this time period. What's more, the three topics of “train ticket,” “Spring Festival transport,” and “return home” appeared frequently in the negative emotion texts. The results may predict that these three topics may be related to the negative emotion of the research group at this stage. In June 2017, the topics of “Hangzhou nanny arson case” appeared frequently. The topic basically coincides with the period of time when the negative emotion of migrant workers appear. Among them, “Hangzhou nanny arson case” is an emergency, and “wage arrears,” “return home,” and “Spring Festival travel” are topics of general concern for this group. This shows that in addition to daily life, this group also pay attention to social affairs. It also shows that the migrant works' emotion can be effectively intervened and guided.

## Conclusion

Migrant workers are a unique social phenomenon in the process of industrialization and urbanization of our country. Their adaptation after entering the city is of great significance to the urbanization and modernization. However, as urban “migratory birds,” the majority of migrant workers drift between urban and rural areas, encounter “marginalization” and rejection from all sides, they will inevitably face emotional dilemmas. In view of this, it is particularly important to understand the ideological trends of migrant workers in a timely manner, to grasp the concerns of migrant workers in an all-round way, and to effectively monitor and analyze their emotion. Only when the country, the government and the whole society timely understand the concerns of migrant workers, and detect signs and tendencies as early as possible, can they formulate relevant policies in a more targeted manner and resolve conflicts as soon as possible.

Based on the web text, the paper has conducted a comprehensive research on the concerns and emotion analysis of migrant workers and achieved good empirical results, but there are still the following aspects that need to be further improved. First of all, in terms of data collection, the paper only collected the data from Weibo. Although the data source after pre-processing is highly credible, the data range is relatively small. The current crawling methods in this paper are only for designated websites, and cannot crawl all the websites of the whole network. In other words, web page analysis is not intelligent enough and comprehensive. Secondly, to further improve the recognition rate of positive and negative emotion, the method of emotion analysis for migrant workers needs to be improved. However, this article only analyzes sentiment from the concerns of migrant workers and wages, which is not enough. Due to the limited information collected by the data set, it is impossible to analyze the emotions of migrant workers more comprehensively. In the future, we intend to collect and use more comprehensive and targeted data sets for emotion analysis.

## Data Availability Statement

The original contributions presented in the study are included in the article/supplementary material, further inquiries can be directed to the corresponding author.

## Author Contributions

ZD conceived and designed the framework of the manuscript. From the determination of the research problem, the selection and implementation of the solution are all done independently by ZD. During the implementation of the plan, both ZD and ZC participated in model training, experimental data evaluation and analysis, and manuscript writing. DH was mainly responsible for controlling the entire process from conception to completion of the manuscript and making suggestions. All authors contributed to the article and approved the submitted version.

## Conflict of Interest

The authors declare that the research was conducted in the absence of any commercial or financial relationships that could be construed as a potential conflict of interest.

## Publisher's Note

All claims expressed in this article are solely those of the authors and do not necessarily represent those of their affiliated organizations, or those of the publisher, the editors and the reviewers. Any product that may be evaluated in this article, or claim that may be made by its manufacturer, is not guaranteed or endorsed by the publisher.
